# Beneficial effects of plant-associated microbes on indoor microbiomes and human health?

**DOI:** 10.3389/fmicb.2014.00015

**Published:** 2014-01-29

**Authors:** Gabriele Berg, Alexander Mahnert, Christine Moissl-Eichinger

**Affiliations:** ^1^Institute of Environmental Biotechnology, Graz University of TechnologyGraz, Austria; ^2^Institute for Microbiology and Archaea Center, University of RegensburgRegensburg, Germany

**Keywords:** interplay of plant -and indoor microbiomes, management of beneficial microbes, next-generation sequencing, houseplants, omics-technologies

## Plant microbiomes—an introduction

Just like humans, plants have recently been recognized as meta-organisms, possessing a distinct microbiome and revealing close symbiotic relationships with their associated microorganisms (Berg et al., 2013; Mendes et al., 2013). Each plant harbor specific species to a certain degree but also cosmopolitan and ubiquitous microbial strains; the majority of them fulfill important host as well as ecosystem functions (rev. in Berg and Smalla, 2009). In addition to the microbe-rich rhizosphere, which has been studied extensively, the phyllosphere is of special interest for the study of indoor microbiomes due to its large and exposed surface area and its remarkable microbial diversity (Lindow and Leveau, 2002; Lindow and Brandl, 2003; Redford et al., 2010; Meyer and Leveau, 2012; Vorholt, 2012; Rastogi et al., 2013). In addition to the majority of beneficial and neutral inhabitants, all plant-associated microbiomes contain plant as well as human pathogens (Berg et al., 2005; Mendes et al., 2013). A broad spectrum of plant pathogens is well-known from disease outbreaks. Human pathogens belong mainly to the so called opportunistic or facultative human pathogens such as *Burkholderia cepacia*, *Pseudomonas aeruginosa* or *Stenotrophomonas maltophilia*, which cause diseases only in patients with predisposition or in hospital (Berg et al., 2005; Ryan et al., 2009).

Microbiomes of humans and plants are currently intensively studied using the same methods and addressing similar scientific questions (Ramírez-Puebla et al., 2013). However, knowledge about the microbiomes' interaction, microbial dynamics and exchange in a certain biotope or even indoor environment is very much limited. Although the composition and function of plant microbiomes is well-studied, there is still little to no information regarding their overlap, interaction with -and impact on other microbiomes or the microbiome-harboring hosts. Information is available about the connection of soil and rhizosphere microbial diversity, which share a selective sub-set (Smalla et al., 2001). The root-soil interface is the selection site for plant-associated bacteria by root exudates, which acts as chemo-attractants as well as repellents to which bacteria respond (Badri and Vivanco, 2009). In addition, plant defense signaling play a role in this process (Doornbos et al., 2012). For the phyllosphere we know that there is only a part of residents, while a substantial part of bacteria is shared with the air microbiome (Lindow and Brandl, 2003). Based on these data, a strong interaction and exchange of rhizosphere and phyllosphere microbiomes with other microbiomes is obvious. However, this opinion paper focuses on the question, if there is also a connection from plant–to indoor microbiomes as well as an impact on human health.

## Indoor microbiomes— importance and origin

Despite the fact that the majority of our lifetime is spent in indoor environments such as home, work place, or public buildings, our knowledge of microbial diversity inside buildings is limited. We are not alone in these indoor environments: they provide new habitats and residence to numerous microbial communities comprising possibly hundreds of individual bacterial, archaeal and fungal species including diverse viruses. Recent studies analyzed potentially pathogenic and allergenic indoor microorganisms with mainly cultivation-based methods (Täubel et al., 2009; Yamamoto et al., 2011). Since the fraction of cultivable microbes on one specific medium is extremely low, information about specifically-adapted micro-organisms, or those with special needs, remains inaccessible by standard cultivation assays. Recently, however, the application of molecular methods, including next generation sequencing (NGS) techniques has provided new insights into indoor microbial communities, revealing a generally high prokaryotic diversity including diverse bacterial, archaeal and fungal phyla (Flores et al., 2011, 2013; Moissl-Eichinger, 2011; Hewitt et al., 2012, 2013; Kembel et al., 2012; Dunn et al., 2013; Kelley and Gilbert, 2013; Meadow et al., 2013).

Indoor microbial communities are an important component of everyday human health (Arundel et al., 1986; Lee et al., 2007; Kembel et al., 2012). Due to human activity and high emission rate of up to 10^6^ bacteria per person-hour as measured via 16S rRNA gene quantification from aerosols (Qian et al., 2012), indoor environments are strongly influenced by typically human-associated bacteria (Fierer et al., 2008). Hence, built environments like hospitals are more easily colonized to a large extent by patient-associated microbes (Oberauner et al., 2013). As a result, many patients in hospitals and especially in intensive care units (ICUs) develop hospital-acquired “nosocomial infections” that compound their underlying severe disease (Vincent et al., 1995; Plowman, 2000). Moreover, these nosocomial infections remain among the leading causes of death in developed country hospitals. The risk to get nosocomial infections for patients in European ICUs was reported as 45% (Plowman, 2000). Hospital surfaces are often overlooked reservoirs for these bacteria (Hota, 2004; Gastmeier et al., 2005; Kramer et al., 2006). Apart from hospitals, indoor microorganisms affect human health as allergenic agents as well (Hanski et al., 2012). Indoor microorganisms are also involved in the development of the Sick Building Syndrome (SBS), which causes symptoms such as sensory irritation of the eyes, nose, and throat, neurotoxic or general health problems, skin irritation, non-specific hypersensitivity reactions, and odor and taste sensations (Godish, 2001).

Indoor microbiomes originate primarily from human skin, pets, or the outside air (Flores et al., 2011; Kembel et al., 2012; Meadow et al., 2013). Plants as a source of indoor microbes are so far less considered. However, air-borne microbes as substantial part—bacteria, fungi or microscopic algae—are scattered and can travel long distances such as in the wind or in clouds before returning to ground-level (Hamilton and Lenton, 1998). They have received more attention because they can serve as nuclei for condensation and as such influence our world climate as rain-making bacteria. Interestingly, cloud and hailstone studies indicated plant-surface bacteria as the dominant source of these rain-making microbes (Morris et al., 2008; Šantl-Temkiv et al., 2013). In addition, little is known about the impact of houseplants and its microbes, although older studies indicate indoor plants as important source (Burge et al., 1982).

**Figure 1:**
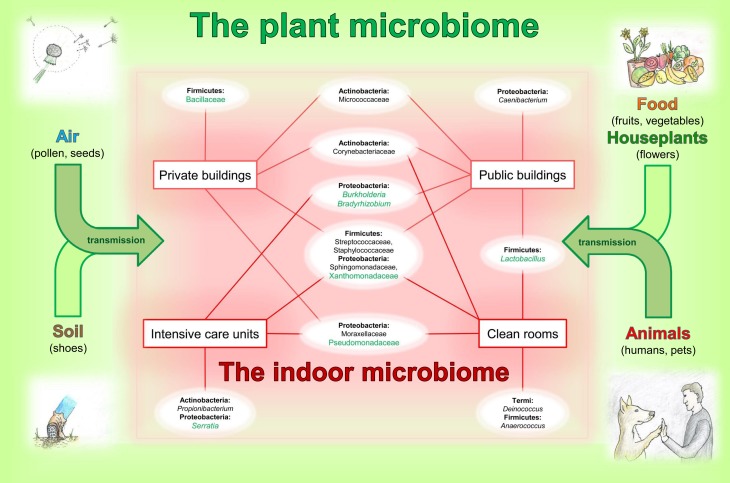
**Relationships between the plant and indoor microbiome**. The indoor microbiomes, influenced by transmissions via air, soil, food, houseplants and animals from plant microbiomes, presents an overview on typical and dominant bacterial groups occurring in the built environment. Schematic chart represents occurrence of the bacterial inhabitants indoors. Bacterial families and genera (white ellipses) are arranged according to their phylum affiliation (bold) and are connected to certain types of the built environments (red squares). Taxa highlighted in green are typical phyla detectable in plant microbiomes. This image has no demand of being complete.

Comparing indoor with plant microbiomes, it is our opinion that both outside and inside plants are of importance for our indoor microbiome. Plants provide beneficial bacteria for indoor rooms and therefore can positively influence human health. The following facts support our opinion about the importance of plants as source for a beneficial microbial biodiversity:

1. Empirically the positive effects of houseplants and flowers are well-known, but there is also evidence for psychological effects such as stress reduction and creative task performance (Fjeld et al., 1998; Shibata and Suzuki, 2004; Chang and Chen, 2005; Bringslimark et al., 2007; Dijkstra et al., 2008). In addition houseplants feature a remarkable capacity to improve indoor air quality (Orwell et al., 2004). This melioration of indoor air is not only due to the filtering capacity of plant leaves, but also by the degrading effects of their root associated microbes (Pegas et al., 2012 up to 90% formaldehyde removal during night according to Kim et al., 2008).
2. Plant DNA as frequently detected as chloroplast 16S rRNA gene sequences in amplicon surveys is a substantial part of all indoor microbiomes, but mainly filtered out for the presentation of data (Oberauner et al., 2013). This emphasizes, that pollen and seeds of plants, which are densely colonized by bacteria (Fürnkranz et al., 2012) are dispersed into the indoor environment and thus provide excellent shuttles for microbiome exchange.
3. Typical and often dominant plant-associated bacteria are members of the indoor microbiome. A relationship of bacteria genera occurring on plants and indoors is given in Figure 1. There are many ways for plant microbes to enter the built environment; as already mentioned on pollen, seeds, fog, soil on shoes, flowers, fruits and vegetables as well as transmitted by animals and other visitors.
4. At species level, no differentiation was possible for clinical and plant-associated isolates. This was studied for *Burkholderia cepacia*, *Pseudomonas aeruginosa* and *Stenotrophomonas maltophilia* (Ryan et al., 2009; Martins et al., 2013). Unfortunately, these plant-associated bacteria can infect immuno-compromised patients with high predisposition in hospitals. On the one hand this is an evidence for the interplay of the plant and indoor microbiome, but on the other hand it highlights the beneficial balance, which is necessary between microorganisms and hosts.
5. Interestingly, Thaumarchaeota, originally described to be associated with ammonia-oxidation in soil and the rhizosphere of plants, have been found on human skin (Probst et al., 2013). Currently it is unknown, whether the human skin archaea have positive or negative effect on human health and whether they have different genomic capabilities compared to their soil-relatives. However, it becomes clear, that closely related microorganisms can exist in different microbiomes, based on a dynamic exchange or distribution and subsequent development of adaptation strategies.

Based on these facts, we speculate the following:

Enclosed environments and their microbiomes—like private/public buildings, hospitals, and clean rooms, which are more or less separated from outside, are especially shaped by human influence and human associated microbes (Hospodsky et al., 2012; Dunn et al., 2013). Hence, microbial diversity is altered and partially reduced compared to the outdoor environment. A reduction in microbial diversity is well known to facilitate dominant proliferations of certain strains, which might bear the risk to have a negative effect toward our health. To increase microbial diversity in an indoor environment we could simply open our windows instead of using air-condition (Hanski et al., 2012; Kembel et al., 2012; Meadow et al., 2013). Alternatively, we could use potted houseplants in built environments as a source of microbial biodiversity and possibly beneficial microorganisms.

Microbes, which live in close vicinity to human beings, are adapted to us as symbionts, commensals, or pathogens, whereas these life-styles are changeable dependent on the host-microbe balance. Indoors we share these microbes, which might get deposited on various surfaces by one person and afterwards get collected by another. Human-associated microbes e.g., skin associated, are confronted with totally new biotic and abiotic factors in the built environment. Here they have to adapt to new surface materials, compete with others for scarce nutrients and withstand stresses associated to cleaning reagents etc. However, in the case of houseplants we allow them to proliferate in a protected environment. Plant associated microbes stay on the leave or stem surface, where they have adapted to and are sheltered from cleaning procedures. Although these phyllosphere communities are confronted with an absence of direct sun light and rain as well as other changed meteorological parameters like air/dust turbulences, their rhizosphere and surrounding soil communities stay in their natural habitat. Hence, these well balanced plant communities, which we bring inside, have the potential to balance an indoor microbiome, by increasing its diversity and filter airborne microbes.

## Conclusion

Members of the plant microbiome are an important source for indoor microbiomes. Both, plants from inside and outside can contribute to the micro-flora. Plant-associated bacteria could act as counterparts against pathogens within the microbial ecosystems. They stabilize the ecosystem, enhance biodiversity and avoid outbreaks of pathogens. However, more research is necessary to understand the microbiology of indoor environments. Currently used cleaning and hygiene strategies in built environments especially in hospitals and ICUs often promote multi-resistant pathogens instead of supporting beneficials. In future, it is important to re-think our understanding of necessary sterility and our relationship to our surrounding microbiomes. This “paradigm shift in ecology” is not only required for plants, humans (Jones, 2013) but also for our environment. Fortunately, “omics”-technologies guided by next-generation sequencing and microscopic techniques allow us now a much better assessment of them. Moreover, we can develop management strategies for beneficial interactions.
